# Immunotherapy in Breast Cancer: the New Frontier

**DOI:** 10.1007/s12609-018-0274-y

**Published:** 2018-04-16

**Authors:** Zishuo I. Hu, Heather L. McArthur

**Affiliations:** 1 Mount Sinai Health System, Icahn School of Medicine, New York, NY USA; 2 Department of Medicine, Breast Oncology, Samuel Oschin Comprehensive Cancer Institute, Cedars-Sinai Medical Center, 8700 Beverly Blvd, 1S35, Los Angeles, CA 90048 USA

**Keywords:** Breast cancer, Immunotherapy, Checkpoint inhibitors, Vaccines

## Abstract

**Purpose of review:**

This review summarizes current immunotherapies in breast cancer, with an emphasis on immune checkpoint inhibitors and vaccines.

**Recent findings:**

Combination immunotherapy with checkpoint inhibitors and cytotoxic therapies have shown promising results. Active clinical trials are ongoing in both early stage and metastatic settings for triple negative, HER2+, and hormone-positive breast cancer patients.

**Summary:**

Ongoing challenges remain in defining biomarkers that predict response to immunotherapy, determining the optimal combination immunotherapies, and enhancing the immunogenicity of breast cancer subtypes.

## Introduction

Immunotherapy has recently proven a promising therapeutic strategy for both hematological and solid malignancies. Although most breast cancers are not inherently immunogenic, recent efforts employing checkpoint inhibitors either as monotherapy or in combination with local or systemic strategies have yielded promising results. Herein, we discuss immunotherapy strategies under investigation for breast cancer, with a focus on immune checkpoint inhibitors and cancer vaccines. We also review future challenges and growing areas of research in the field of breast cancer immunotherapy.

## Immune Checkpoint Inhibitors

Immune checkpoint inhibitors enable T cell activation by agonizing costimulatory signals or antagonizing co-inhibitory signals. Specifically, T cell inhibition is interrupted with the administration of antibodies that bind to cytotoxic T lymphocyte antigen 4 (CTLA-4) or programmed death-1 or its ligand (PD-L1), thereby permitting robust and sustained T cell responses. T cell activation is initiated with the presentation of antigens to the major histocompatibility complex (MHC) molecule on the surface of antigen presenting cells (APCs). After binding of the T cell receptor (TCR) to the MHC-antigen complex, the B7 protein on the APC binds to the T cell costimulatory molecule CD28 to promote T cell activation and survival. Inhibition of T activation is mediated by the subsequent upregulation of CTLA-4 on T cells, which competes with CD28 to bind B7. Further control of T cells response in peripheral tissue is regulated by the expression of PD-1 on activated T cells. The binding of PD-1 to its ligand PD-L1 induces an inhibitory signal that limits T cell proliferation and survival [1–3].

### CTLA-4 Inhibitors

One of the first clinical trials to administer checkpoint inhibitor therapy in breast cancer was a phase 1 study using the monoclonal fully human CTLA-4 antibody, tremelimumab, in combination with exemestane in 26 post-menopausal women with metastatic hormone-positive or hormone-responsive breast cancer [4]. The best reported overall response (ORR) was durable, stable disease in 11 of the 26 patients (42%). The majority of the treatment-related adverse events (AE) were mild to moderate, with the most common being diarrhea (46%) and pruritus (42%). Furthermore, 7 (27%) had grade 3 AEs, with one serious AE and no grade 4 AEs. Notably, treatment was also associated with an increase in the percent of activated CD4+ and CD8+ T cells as measured by inducible costimulatory (ICOS) expression and marked increase in the ratio of peripheral activated T cells to suppressive regulatory T cells. More recently, CTLA4-blockade has been explored in combination with local strategies such as tumor freezing, or cryoablation, in the curative-intent setting (see below) [5, 6].

#### PD-1/PD-L1 Inhibitors

##### PD-1/PD-L1 Inhibitors as Monotherapy for Metastatic TNBC

One of the first reports of PD-1/PD-L1 inhibition in breast cancer was a phase 1b trial of the anti-PD-1 antibody pembrolizumab in 32 women with PD-L1-“positive” metastatic triple negative breast cancer (mTNBC) (KEYNOTE-012) [7]. In this study, PD-L1 positivity was defined as PD-L1 expression in the stroma or in ≥ 1% of tumor cells. The overall response rate was 18.5% in 27 evaluable patients, and the median time to response was 17.9 weeks (range, 7.3 to 32.4 weeks). Median overall survival (OS) was 10.2 months, and the 12-month OS rate was 41.1%. A total of 15.6% of the patients had at least one grade 3 to 5 AE, with the most common AE being arthralgia (18.8%) and fatigue (18.8%). Of the five responders, one had a complete response (CR), four had partial responses (PRs), and three have had long-lasting benefit from pembrolizumab. At the time of publication, median duration of response had not yet been reached.

In a phase 1b study, the anti-PD-L1 antibody, atezolizumab, was evaluated in women with PD-L1-“positive” metastatic TNBC [8]. The PD-L1 tumor-infiltrating immune cell (IC) status was defined by the percentage of PD-L1-positive ICs: IC 0/1 < 5% or negative and IC 2/3 (≥ 5%) or positive. Of the response evaluable population (*N* = 21), the ORR was 19%. Of the safety evaluable population (*N* = 54), 11% of the patients had at least one grade 3 to 5 AE. This trial was later expanded to include patients regardless of PD-L1 status [9]. In the expansion, the ORR for the 112 evaluable patients was 10% overall. Patients with high PD-L1-expressing tumors had higher ORRs compared to patients with low PD-L1 expression (13 versus 5%, respectively). The ORR was 26% for patients treated with atezolizumab in the first-line versus 4% in the second-line setting with a 21-month median duration of response which is remarkable for this generally poor prognosis population.

In the JAVELIN study, the PD-L1-directed antibody avelumab demonstrated modest responses across breast cancer subtypes with an ORR of 8.6% in the TNBC cohort unselected for PD-L1 expression [10]. Notably, there was no impact on response by various tumor PD-L1 cut-offs.

In KEYNOTE-086, pembrolizumab was administered as either salvage treatment for metastatic TNBC patients regardless of PD-L1 expression (cohort A) or as first-line therapy for patients with metastatic PD-L1+ TNBC (cohort B) [11, 12]. The ORR was 4.7% in the 170 previously treated patients (cohort A), with no significant difference observed by PD-L1 expression. Median OS was 8.9 months. The ORR for the 52 previously untreated patients with PD-L1-positive TNBC (cohort B) was 23%. Thus, 23–26% of women with TNBC respond to PD-1 or PD-L1 blockade in the first-line setting. However, because very modest responses are observed with monotherapy in the second-line setting and beyond, combination strategies are needed if the majority of immunologically “cold” tumors are to be converted into immune-responsive tumors.

##### PD-1/PD-L1 Inhibitors in Combination with Chemotherapy for mTNBC

The combination of chemotherapy with PD-1/PD-L1-targeted therapies has shown promising results in mTNBC. For example, in a phase 1b study of 32 patients with evaluable mTNBC, the combination of atezolizumab and nab-paclitaxel resulted in an ORR of 38%, with similar response rates in the first versus third line and beyond (46 versus 40%, respectively) [13]. The ORR by PD-L1 expression was 30% for IC0, 36% for IC1/2/3, and 46% for unknown expression, respectively. A phase 3 multicenter, randomized, double-blind placebo-controlled trial (IMpassion130, NCT02425891) is ongoing to assess nab-paclitaxel with or without atezolizumab as first-line treatment in mTNBC.

In phase 1 study of eribulin with pembrolizumab in mTNBC, an ORR of 34% was reported overall, with an ORR of 41% in an interim analysis of 17 patients treated in the first-line setting versus 27% in the 2nd and 3rd line settings [14]. Consistent with the nab-paclitaxel with atezolizumab experience, no significant differences in responses were observed by PD-L1 expression in the eribulin with pembrolizumab study. Moreover, across studies, when responses occur, they tend to be durable.

##### PD-1/PD-L1 Inhibitors in Combination with Chemotherapy for Early Stage TNBC

Given the encouraging results observed with chemotherapy/checkpoint blockade combinations in the mTNBC setting, there is considerable interest in applying these strategies with curative intent. The I-SPY2 trial has an adaptive randomization design that allows for expeditious evaluation of drugs in the pre-operative setting to determine whether they are likely to be successful in a randomized study. In a recent report from I-SPY2, paclitaxel was administered with or without pembrolizumab followed by four cycles of conventional doxorubicin with cyclophosphamide (AC) in women with early stage HER2-normal disease in the pre-operative setting [15]. When pembrolizumab was added to standard chemotherapy, the estimated pathologic complete response rate (pCR) was approximately 20% in the control arm versus 60% in the pembrolizumab-containing arm for the subset of women with TNBC. Although the estimated pCR rate in the control arm is less than expected for pre-operative chemotherapy with a taxane and anthracycline in TNBC, the magnitude of the difference across the arms was striking. However, the addition of pembrolizumab to the chemotherapy backbone added to the toxicity of the regimen with a rate of adrenal insufficiency of 8.7 versus 0% for the pembrolizumab versus control arms, respectively. In KEYNOTE-173, a phase 1b trial of neoadjuvant pembrolizumab with anthracycline and taxane-based chemotherapy in TNBC, a pCR rate of 80 versus 50% was achieved with or without the addition of carboplatin [16]. However, the improvement in pCR with the addition of carboplatin was associated with an 80% grade 3/4 neutropenia rate. Thus, close safety monitoring is critical as these combination strategies are being explored in the curative-intent setting. There are currently several large randomized studies planned or underway. For example, in KEYNOTE-522, women with stage II/III TNBC are being randomized at diagnosis to receive neoadjuvant chemotherapy (including an anthracycline, taxane, and carboplatinum) with or without pembrolizumab (NCT03036488) [17]. In Southwest Oncology Group (SWOG) 1418, patients with residual TNBC after neoadjuvant chemotherapy are randomized to a year of pembrolizumab versus observation (NCT02954874). And in IMpassion030, women with stage II/III TNBC will be randomized to anthracycline and taxane-based adjuvant chemotherapy with or without a year of adjuvant atezolizumab (NCT03197935). It is hoped that these chemotherapy-checkpoint blockade combination strategies will improve cure rates and potentially permit de-escalation of cytotoxic backbones.

#### PD-1/PD-L1 Inhibitors in Hormone Receptor-Positive, HER2-Negative Disease

KEYNOTE-028 was a phase 1b multicohort study of pembrolizumab for PD-L1-positive advanced tumors [18]. In this study, PD-L1 positivity was again defined as PD-L1 expression in the stroma or in ≥ 1% of tumor cells. Among the 25 patients with estrogen-positive/HER2-negative metastatic breast cancer treated on study, an ORR of 12% was reported with three PRs with only 16% of patients experiencing a grade 3 or grade 4 AE. More modest response rates were observed in the phase 1b JAVELIN study, wherein 72 women with hormone receptor-positive, HER2-negative disease were treated with avelumab irrespective of PD-L1 expression [10]. In this subset, an ORR of 2.8% was observed. However, in the I-SPY2 study, wherein pre-operative chemotherapy was administered with or without pembrolizumab in a curative-intent population, the estimated pCR rate was 34 versus 13%, respectively, in women with hormone receptor-positive/HER2-normal breast cancer [15]. Thus, although hormone receptor-positive disease may not be innately sensitive to checkpoint blockade monotherapy, chemotherapy combinations—particularly when administered earlier in the course of disease—may be particularly effective.

#### PD-1/PD-L1 Inhibitors in HER2-Positive Disease

Trastuzumab has been shown to have immune-mediated mechanisms of action [19, 20], and pre-clinical studies have demonstrated synergy with checkpoint blockade in combination with HER2-directed therapy [21]. To date, however, there is a paucity of data exploring checkpoint blockade in this subset. In the phase 1b JAVELIN study, one response (3.8%) was observed among the 26 women with metastatic HER2-positive disease treated with avelumab [10].

In PANACEA, 12 women with HER2-positive, PD-L1-negative, and 40 women with HER2-positive, PD-L1-positive disease with prior progression on trastuzumab received concurrent trastuzumab with pembrolizumab in the phase II portion of the study [22]. The best overall response was 15% in the PD-L1-positive and 0% in the PD-L1-negative cohorts. Several studies exploring rational combinations of HER2-directed therapy with checkpoint blockade in the palliative- and curative-intent setting are planned or underway.

#### Combinations of Local Strategies (Cryoablation or Radiation) with Checkpoint Blockade

In a pilot study, the CTLA-4-directed antibody, ipilimumab, versus primary tumor cryoablation versus the combination was explored prior to mastectomy in 19 women with early stage breast cancer [5]. Specifically, 7 women received tumor cryoablation alone, 6 women received a single-dose of ipilimumab alone, and 6 women received the combination of tumor cryoablation and ipilimumab. Cryoablation and ipilimumab (at 10 mg/kg) were safe alone and in combination, with no treatment-associated Grade 3/4 AEs reported. The combination was also associated with increased levels of activated and proliferating CD4+ and CD8+ T cells, and post-treatment proliferative T-effector cells. Furthermore, ipilimumab alone increased intratumoral T cell density over time, whereas cryoablation with or without ipilimumab resulted in clonal expansion of T cells as demonstrated by deep sequencing of T cell receptor DNA [6]. A follow-up pilot study (NCT02833233) evaluated the combination of cryoablation, ipilimumab, and the PD-1 antibody nivolumab in the pre-operative setting, and the results of that effort are expected to directly inform a large randomized study.

Because radiation is immunogenic and can optimize antigen presentation, combinations of radiation with checkpoint blockade are being explored. A study of standard-of-care brain radiation with tremelimumab-mediated CTLA4 blockade for women with breast cancer brain metastases conferred non-CNS disease control in 10% (2/20) of women with HER2-normal disease [23]. However, tremelimumab co-administered with trastuzumab and brain radiation in women with HER2-positive disease conferred non-CNS disease control in 33% (2/6). In a study combining non-CNS radiation with pembrolizumab in women with TNBC, 3 of 9 (33%) evaluable women at 12 weeks had a partial response outside of the radiation field [24]. A similar study combining radiation with pembrolizumab in hormone receptor-positive metastatic breast cancer is underway (NCT03051672).

The TONIC trial (NCT02499367) is a phase 2 randomized trial that aimed to explore the issue of priming for checkpoint blockade. Specifically, nivolumab was administered after induction therapy with radiation (3 × 8 Gy of radiation to one metastatic lesion), low-dose chemotherapy (doxorubicin weekly, cyclophosphamide daily, or cisplatin weekly for 2 weeks), or no induction therapy in 50 patients with mTNBC [25]. Initial results reported an ORR of 10% in patients treated with RT, 45% with doxorubicin, 11% with cyclophosphamide, 33% with cisplatin, and 11% with no induction. Thus, this small study underscores the importance of dose, schedule, and sequence whenever checkpoint blockade is combined with systemic or local strategies.

## Cancer Vaccines in Breast Cancer

A number of cancer vaccine strategies have been tested in breast cancer, including monovalent vaccines, polyvalent vaccines, and cellular vaccines. Monovalent vaccines provide a single tumor-associated antigen (TAA) target for the immune system. Polyvalent peptide vaccines provide multiple TAA targets, and cellular vaccines are ex vivo modified tumor cells or APCs.

### Monovalent Vaccines

Targets used in monovalent vaccines for breast cancer include HER2, mucin 1 (MUC1), and carcinoembryonic antigen (CEA). The E75 peptide vaccine, nelipepimut-S, is derived from the extracellular domain of the HER2 protein. In clinical trials, it has been tested with granulocyte-macrophage colony-stimulating factor (GM-CSF) and has been found to be safe in phase I and II clinical trials. GP2 is an MHC Class I peptide derived from the transmembrane domain of the HER2 protein. The GP2 peptide combined with GM-CSF found the vaccine to be safe and capable of generating a HER2-specific T cell response in early phase trials. A subsequent phase II study with 180 patients was performed with an intention-to-treat analysis performed at a median follow-up of 34 months [26]. The analysis showed an 88% estimated 5-year disease-free survival (DFS) rate in vaccinated patients versus 81% in GM-CSF-only patients (*p* = 0.43). Treatment analysis, which excluded recurrences during the primary vaccination series and secondary malignancies, showed 100% DFS in vaccinated HER2-positive patients compared with 89% in GM-CSF-treated only patients (*p* = 0.08).

MUC1 is a glycoprotein normally expressed on the cell surface of epithelial cells in the breast, stomach, lung, and urinary tracts. It has been found to be overexpressed in all breast cancers and its epitopes CA 15-3 and CA 27-29 serve as breast tumor markers. The MUC1 epitope, sialyl-Tn (STn), was conjugated with the keyhole limpet hemocyanin (KLH) carrier protein, to form the cancer vaccine Theratope. Despite promising pre-clinical and phase II clinical trials, a multicenter, randomized, phase III trial that consisted of 1028 patients with metastatic breast cancer found no significant differences in median time to progression (TTP) or overall survival time between patients who received the vaccine compared with patients who received the KLH carrier protein alone [27].

### Polyvalent Vaccines

PANVAC (pancreatic vaccine) is a recombinant poxviral vaccine expressing MUC1, CEA, and three T cell costimulatory molecules. A pilot trial that included 12 metastatic breast cancer patients resulted in four patients with stable disease and one patient with a complete response to therapy [28]. A subsequent phase II trial combining PANVAC with docetaxel revealed a median PFS of 7.9 months in patients treated with docetaxel and PANVAC compared to 3.9 months in patients treated with docetaxel alone (*p* = 0.09) [29].

### Cellular Vaccines

The GVAX breast vaccine is an allogeneic, HER2-targeted GM-CSF-secreting cellular vaccine. It was evaluated in combination with a range of a low-dose cyclophosphamide and doxorubicin in 28 patients with metastatic breast cancer [30]. Low-dose cyclophosphamide was able to selectively induce the apoptosis of regulatory T cells compared to effector T cells [31]. The vaccine was also given in combination with trastuzumab and low-dose cyclophosphamide to 20 patients with metastatic HER2+ breast cancer [32]. The authors reported an increase in immune response markers to the vaccine, including polyfunctional HER2-specific CD8+ T cells and delayed type hypersensitivity.

## Conclusions

The clinical experience with immunotherapy for the treatment of breast cancer has grown rapidly in recent years. Checkpoint blockade has been most effective as monotherapy in the first-line mTNBC setting and in combination with chemotherapy in pre-treated mTNBC. However, checkpoint blockade is particularly promising in the curative-intent setting across tumor types. Checkpoint blockade in breast cancer has been associated with tolerable safety profiles, and among responders, shown to have durable responses that compare favorably to standard chemotherapy.

One ongoing challenge is defining the patient population that would most benefit from immunotherapy with checkpoint blockade and/or vaccines. For example, trials of checkpoint blockade have demonstrated mixed results with respect to PD-L1 expression as a predictive marker in breast cancer. Tumor-infiltrating lymphocytes have been more robust as a predictive biomarker across multiple studies of multiple agents; however, reliable and reproducible predictive biomarkers are still needed. Mutational burden, CD8+ T cell density, and oncogenic mutations are all markers currently under scrutiny and evaluation [33]. Combination therapy may serve to act as an equalizing agent for patients with lower expression levels of PD-L1, promoting tumor immunogenicity and increasing response rates. Further research into identifying predictive markers in the setting of a growing number of combinatorial therapy trials is of particular clinical relevance both for checkpoint blockade and vaccine-based strategies. It is certain that as more combinations with immunotherapy are explored, the successful identification of rational therapeutic partners will be paramount.
